# Human pluripotent stem cell (PSC)-derived mesenchymal stem cells (MSCs) show potent neurogenic capacity which is enhanced with cytoskeletal rearrangement

**DOI:** 10.18632/oncotarget.9947

**Published:** 2016-06-11

**Authors:** Kai-Yen Peng, Yu-Wei Lee, Pei-Ju Hsu, Hsiu-Huan Wang, Yun Wang, Jun-Yang Liou, Shan-Hui Hsu, Kenneth K. Wu, B. Linju Yen

**Affiliations:** ^1^ Department of Life Science, National Central University, Jhongli, Taiwan; ^2^ Regenerative Medicine Research Group, Institute of Cellular and System Medicine, National Health Research Institutes (NHRI), Zhunan, Taiwan; ^3^ Center for Neuropsychiatric Research, NHRI, Zhunan, Taiwan; ^4^ Institute of Polymer Science and Engineering, National Taiwan University, Taipei, Taiwan; ^5^ Graduate Institute of Basic Medical Sciences, China Medical University, Taichung, Taiwan

**Keywords:** mesenchymal stem cells (MSCs), human pluripotent stem cells (PSC), human embryonic stem cells (ESCs), induced pluripotent stem cells (iPS), Rho A kinase (ROCK)

## Abstract

Mesenchymal stem cells (MSCs) are paraxial mesodermal progenitors with potent immunomodulatory properties. Reports also indicate that MSCs can undergo neural-like differentiation, offering hope for use in neurodegenerative diseases. However, *ex vivo* expansion of these rare somatic stem cells for clinical use leads to cellular senescence. A newer source of MSCs derived from human pluripotent stem cells (PSC) can offer the ‘best-of-both-worlds’ scenario, abrogating the concern of teratoma formation while preserving PSC proliferative capacity. PSC-derived MSCs (PSC-MSCs) also represent MSCs at the earliest developmental stage, and we found that these MSCs harbor stronger neuro-differentiation capacity than post-natal MSCs. PSC-MSCs express higher levels of neural stem cell (NSC)-related genes and transcription factors than adult bone marrow MSCs at baseline, and rapidly differentiate into neural-like cells when cultured in either standard neurogenic differentiation medium (NDM) or when the cytoskeletal modulator RhoA kinase (ROCK) is inhibited. Interestingly, when NDM is combined with ROCK inhibition, PSC-MSCs undergo further commitment, acquiring characteristics of post-mitotic neurons including nuclear condensation, extensive dendritic growth, and neuron-restricted marker expression including NeuN, β-III-tubulin and Doublecortin. Our data demonstrates that PSC-MSCs have potent capacity to undergo neural differentiation and also implicate the important role of the cytoskeleton in neural lineage commitment.

## INTRODUCTION

The discovery of a stem cell population in the adult brain has opened the possibility of using such tissue-specific stem cells for therapy in neuro-degenerative and ischemic diseases, including Parkinson's disease and stroke [1]. As exciting as these findings are, the rarity and difficulty in procurement of such neural stem cells (NSCs) are significant obstacles to prevalence use of these stem cells [2]. Thus, there is continued interest in finding more accessible sources of human stem cells for application in neurological diseases.

A number of studies have shown that mesenchymal stem cells (MSCs) harbor neural differentiation potential and have therapeutic relevance for treatment of related diseases [3]. Multilineage MSCs were first isolated from the bone marrow (BM) and have subsequently been found in various post-natal tissues [4, 5]. In addition to trilineage paraxial mesodermal differentiation capacity, MSCs have significant immunomodulatory properties, making these adult stem cells highly versatile for broad clinical application [6]. However, these rare post-natal stem cells often require vigorous *ex vivo* expansion to reach the volumes necessary for therapeutic use, and this can result in replicative senescence and functional decline [7]. To overcome this issue, MSCs have been derived from human pluripotent stem cells (PSC) including human embryonic stem cells (ESCs) and induced pluripotent stem cells (iPS), and these PSC-MSCs exhibit similar differentiation capacity and surface marker profile to BM-MSCs [8–10]. The differentiation of PSC to a somatic stem cell type such as MSCs allows for clinical use, since worries of teratoma formation is abrogated when PSCs lose pluripotency [11]. Moreover, PSC-MSCs have the added advantage of being derived from a renewable source since PSCs can theoretically be expanded indefinitely, unlike BM-MSCs and other tissue-specific MSCs which must be continually isolated from primary tissue [12]. Being derived directly from PSC, PSC-MSCs are also at a much earlier stage developmentally than adult BM-MSCs, and can possibly have broader differentiation capacity as has been reported for fetal-stage MSCs [13].

Traditionally, induction of neural differentiation has involved the use of biochemical factors in serum-free conditions [14]. Recent studies, however, show that cytoskeletal rearrangement can efficiently induce a neural-like phenotype in MSCs [15, 16]. In this report, we found that PSC-MSCs express higher NSCs-related genes at baseline, and can efficiently differentiate into neuron-restricted progenitors (NRPs) with inhibition of Rho-associated protein kinases (ROCKs), key molecules in modulation of the cytoskeleton [17]. Our data implicate the use of PSC-MSCs in application towards neuro-related diseases, as well as the importance of the cytoskeleton in neural lineage commitment.

## RESULTS

### PSC-MSCs express higher levels of neural stem cell (NSC)-associated genes at baseline than BM-MSCs

**Figure 1:**
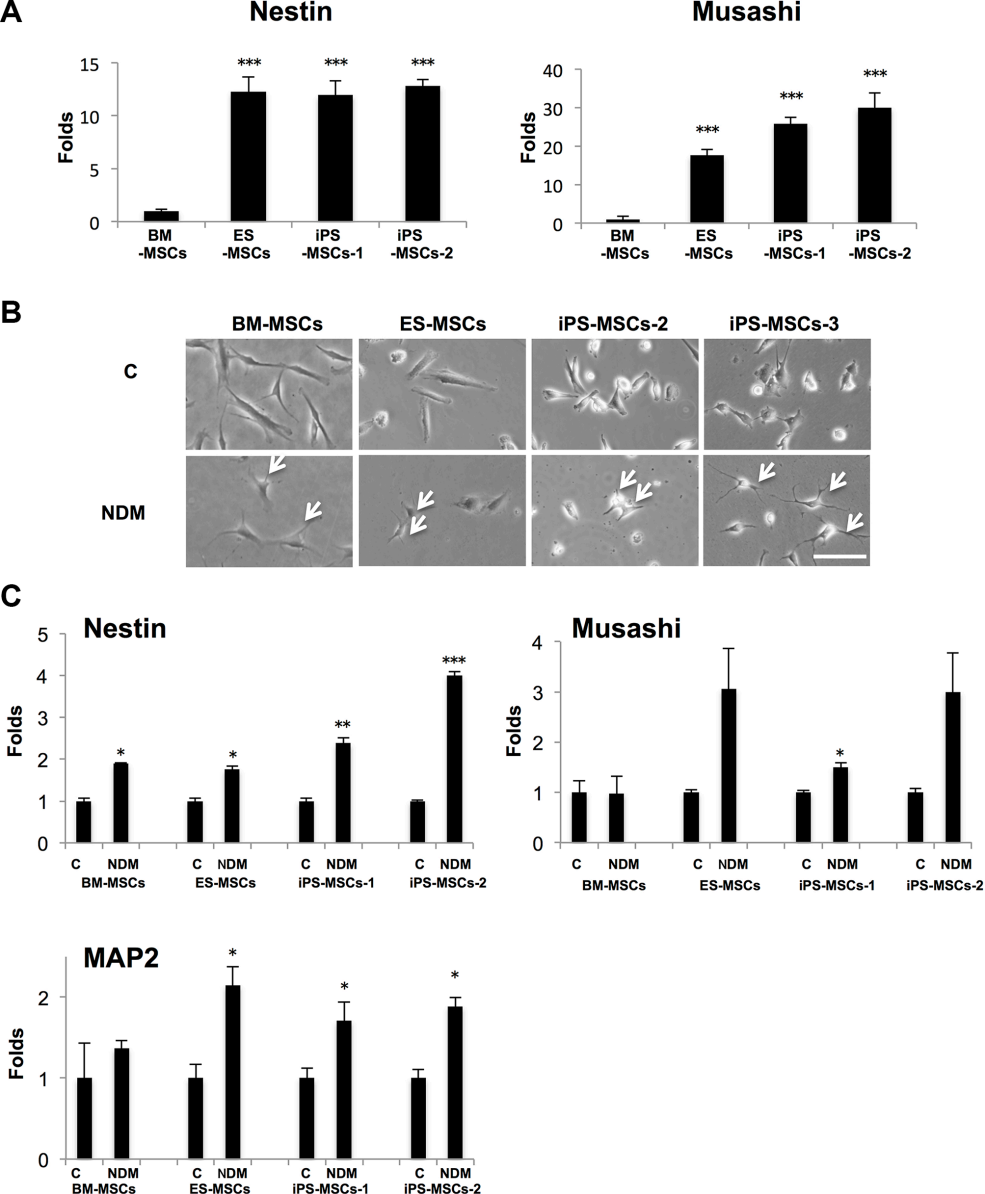
Human pluripotent stem cell-derived mesenchymal stem cells (PSC-MSCs) express higher level of neural stem cell-associated genes than bone marrow mesenchymal stem cells (BM-MSCs) (**A**) Gene expression of *Nestin* and *Musashi* in PSC-MSCs (ES-MSCs, iPS-MSCs-1, and iPS-MSCs-2) and BM-MSCs was analyzed by real-time PCR (qPCR). ***p < 0.005; compared to BM-MSCs. (**B**) Phase-contrast microscopy of BM-MSCs and various PSC-MSCs after being cultured 48 hours in neural differentiation medium (NDM; consist of RA (0.5 μM) in serum-free medium). Arrow indicates dendrite-like processes. (**C**) Gene expression of *Nestin*, *Musashi*, and *MAP2* in PSC-MSCs and BM-MSCs after culturing in control complete medium (C) and NDM for 48 hours as analyzed by qPCR. *p < 0.05 and **p < 0.01, compared to Control. Error bars represent ± SEM of three independent experiments. Scale bar: 20 μm.

To assess the neurogenic potential of human PSC-MSCs, including human ESCs-derived MSCs (ES-MSCs) and iPS-derived MSCs (iPS-MSCs), we performed real-time PCR to detect baseline expression of *Nestin*, an intermediate filament, and *Musashi*, a RNA-binding protein, which are neural stem cell (NSC)-associated genes [18]. We found that at baseline, PSC-MSCs express significantly higher levels of *Nestin* and *Musashi* than adult BM-MSCs (Figure 1A). ES-MSCs (12.3-fold), iPS-MSC-1 (11.9-fold), and iPS-MSC-2 (12.8-fold) express *Nestin* at a level that is on average over 10-fold higher than BM-MSCs. The expression levels of *Musashi* were up to 30.0-fold higher in ES-MSCs (17.6-fold), iPS-MSCs-1 (25.9-fold), and iPS-MSC-2 (30.0-fold) compared to BM-MSCs. We then performed neural differentiation using standard neurogenic differentiation medium (NDM), which consist of serum-free medium with addition of retinoic acid (RA) [14]. After culturing in NDM for two days, BM-MSCs and PSC-MSCs acquired a neural-like morphology with elongated cytoplasmic processes and numerous dendrite-like processes (Figure 1B). NDM also increased the expression of *Nestin*, *Musashi*, and *MAP2* (microtubule-associated protein 2)—a critical protein involved in neural differentiation [19]—in all MSCs, with a more significant increase in PSCs-MSCs compared to BM-MSCs (Figure 1C). These results suggest PSC-MSCs may have a higher neurogenic potential and are more sensitive to neurogenic differentiation signals than BM-MSCs.

### PSC-MSCs acquired an early-stage neural cell phenotype through inhibition of Rho a kinase-myosin II pathway

**Figure 2:**
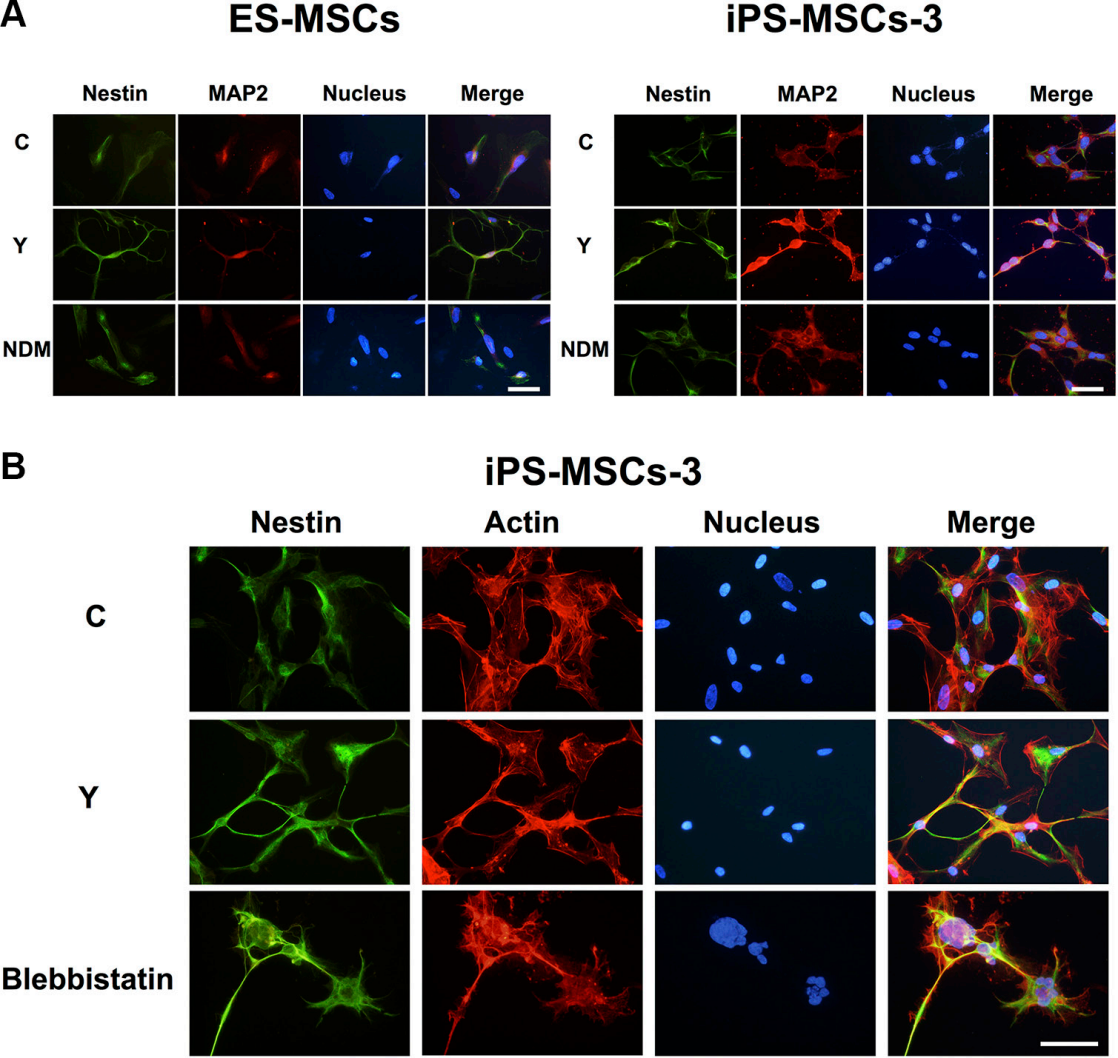
PSC-MSCs express early neural markes after RhoA kinase (ROCK) inhibition (**A**) Immunofluorescent (IF) staining was performed to analyze for Nestin (green fluorescence) and MAP2 (red fluorescence) expression in PSC-MSCs after NDM or complete medium with ROCK inhibition using Y-27632 (Y). Hoechst 33342 was used to detect nuclei (blue). (**B**) Expression levels of Nestin (green fluorescence) and F-actin (red fluorescence) were analyzed by IF staining in PSC-MSCs after blebbistatin treatment. Scale bar: 20 μm.

Cytoskeletal rearrangement in post-natal MSCs has been shown to direct neural lineage commitment through RhoA kinase (ROCK), one of the most important mediators in cytoskeletal dynamics [15, 16]. We sought to ascertain whether cytoskeletal rearrangement in PSC-MSCs could also lead to neural lineage commitment. To address this question, we treated PSC-MSCs cultured in complete medium with Y-27632, a selective inhibitor of ROCK, then assessed for protein expression of neural lineage markers by immunofluorescent (IF) staining. Addition of Y-27632 in complete medium not only promoted a neuron-like morphology but also increased Nestin and MAP2 expression in PSC-MSCs to a similar extent compared to culturing in NDM (Figure 2A). It has been reported that the actions of ROCK inhibition in neural differentiation of MSCs involve myosin II, one of its downstream targets [20]. Correspondingly, when PSC-MSCs are treated with blebbistatin, a specific inhibitor of myosin II ATPase, the morphological changes seen with ROCK inhibition are recapitulated [15, 21]. To assess cytoskeletal dynamics during PSC-MSC differentiation, we stained for F-actin and found that PSC-MSCs treated with Y-27632 and blebbistatin show actin-rich extensions at the leading edge (Figure 2B), a characteristic of axonal and dendritic growth during neuronal development [22]. In contrast, in untreated PSC-MSCs, F-actin remained cytoplasmic. Thus, inhibition of the ROCK-myosin II axis induces neural lineage commitment in PSC-MSCS.

### ROCK inhibition of iPS-MSCs cultured in NDM induce further neural commitment

**Figure 3:**
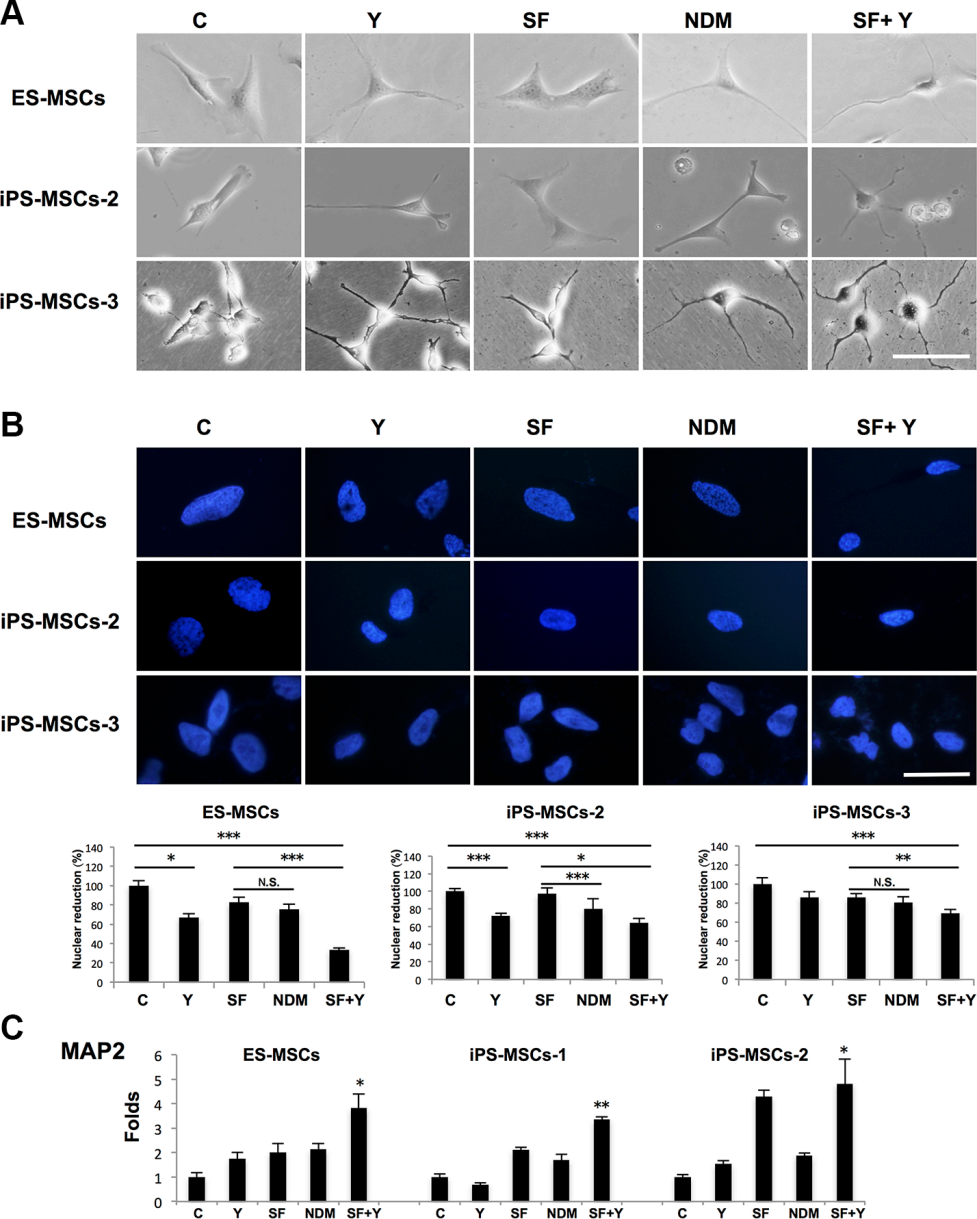
ROCK inhibition in serum-free (SF) conditions induce further neural lineage commitment of PSC-MSCs (**A**) Phase-contrast microscopy of PSC-MSC cell morphology after ROCK inhibition with Y-27632 in complete medium (Y), NDM, and Y-27632 in serum-free medium (SF+Y). (**B**) Nuclear size of PSC-MSCs after culturing in Y, NDM, and SF+Y as quantified by Image J software. Nuclei were be detected by Hoechst 33342 (blue fluorescence). *p < 0.05; **p < 0.01; ***p < 0.005; N.S., not significant. (C) Gene expression of MAP2 in PSC-MSCs after culturing in Y, NDM, and SF+Y as quantified as analyzed by qPCR. All results are shown as mean ± SEM of three independent experiments. *p < 0.05; **p < 0.01; compared to control. Scale bar: 20 μm.

Given the strong baseline expression of NSC-related genes and response to cytoskeletal manipulation, we were interested whether PSC-MSCs could further undergo differentiation into more committed neural lineage cells. NSCs are known to further differentiate into committed cell types: neuron-restricted progenitors (NRPs) and glial-restricted progenitors (GRPs) [23, 24]. To explore whether PSC-MSCs can further differentiate into these more committed neural cells, we cultured cells in serum-free medium—a typical method to induce neural differentiation—with the addition of Y-27632 (SF+Y) [14]. Bright field microscopy showed that PSC-MSCs acquired more committed neural lineage morphology after SF+Y treatment (Figure 3A). During the neural differentiation process from pluripotent cells into post-mitotic neurons, the nucleus undergoes remodeling with concomitant decreases in size; this has been used as an indicator on the extent of neural differentiation [25]. We therefore measured the nuclear area of PSC-MSCs after various neural induction methods, and found that while Y-27632 in complete medium diminished the nuclear sizes of PSC-MSCs, the most significant reduction in nuclear size occurred when PSC-MSCs were cultured in SF+Y especially for ES-MSCs (Figure 3B; average percentage of nuclear reduction: ES-MSCs from 100% down to 33.3%, iPS-MSCs-2 from down to 64.0 %, and iPS-MSCs-3 down to 69.6 %). Furthermore, real-time PCR analysis for expression of *MAP2*, which is a marker of NRPs [26], was also most significantly increased after SF+Y treatment and most apparent in ES-MSCs as well (Figure 3C; average increases in ES-MSCs up to 3.8-fold, iPS-MSCs-1 up to 3.4-fold, iPS-MSCs-2 up to 4.8-fold). Thus, cytoskeletal manipulations in standard neurogenic differentiation conditions can further induce PSC-MSCs towards a more committed neural phenotype.

### PSC-MSCs express NRP-associated proteins after ROCK inhibition in SF conditions

**Figure 4:**
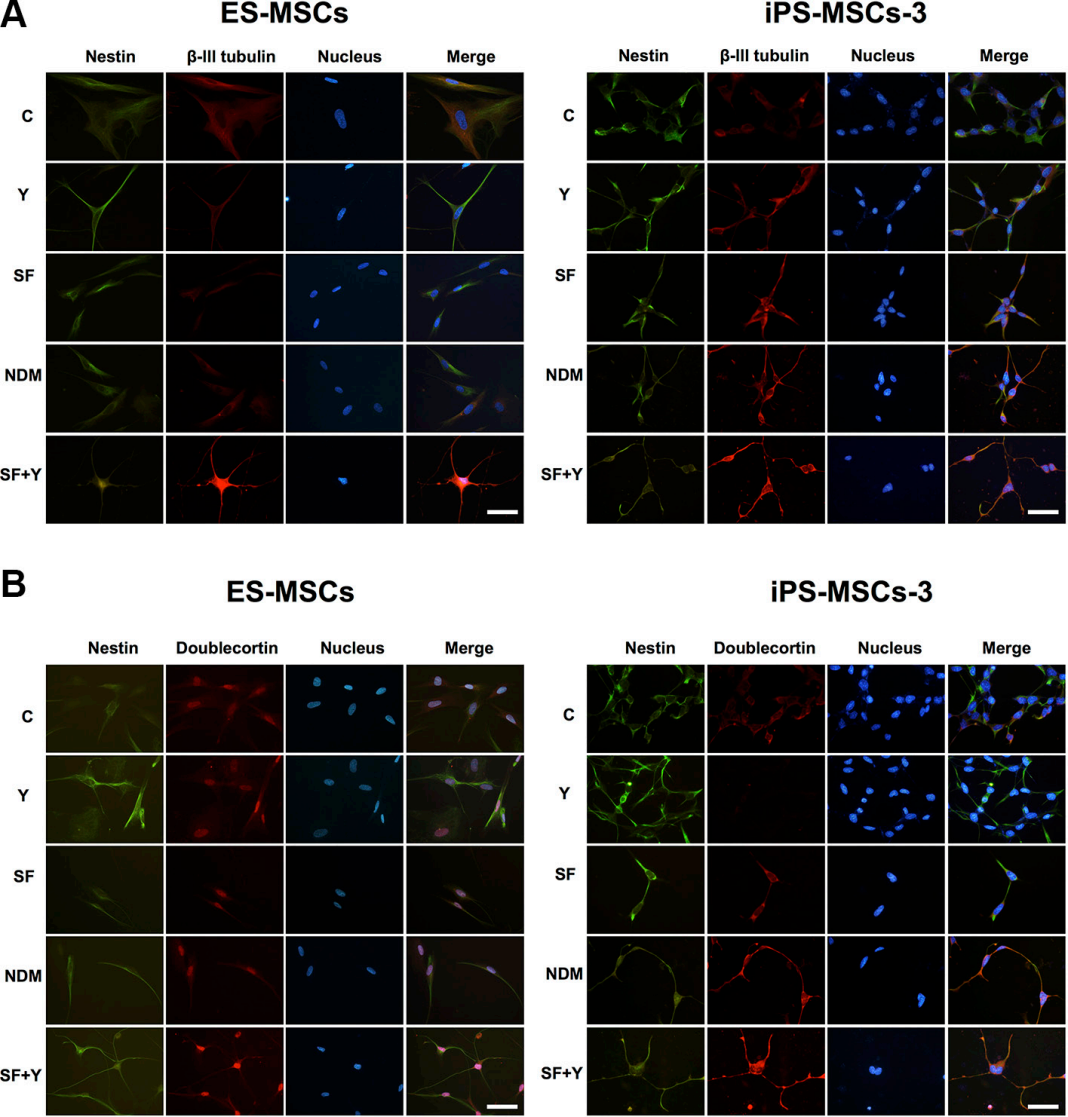
PSC-MSCs express committed neural lineage proteins after ROCK inhibition in SF conditions Protein expression of (**A**) Nestin (green fluorescence) and β-III-tubulin (red fluorescence), or (**B**) Nestin (green fluorescence) and Doublecortin (red fluorescence) in PSC-MSCs after culturing in Y, NDM, and SF+Y as analyzed by IF staining. Nuclei were be detected by Hoechst 33342 (blue fluorescence). Scale bar: 20 μm.

**Figure 5:**
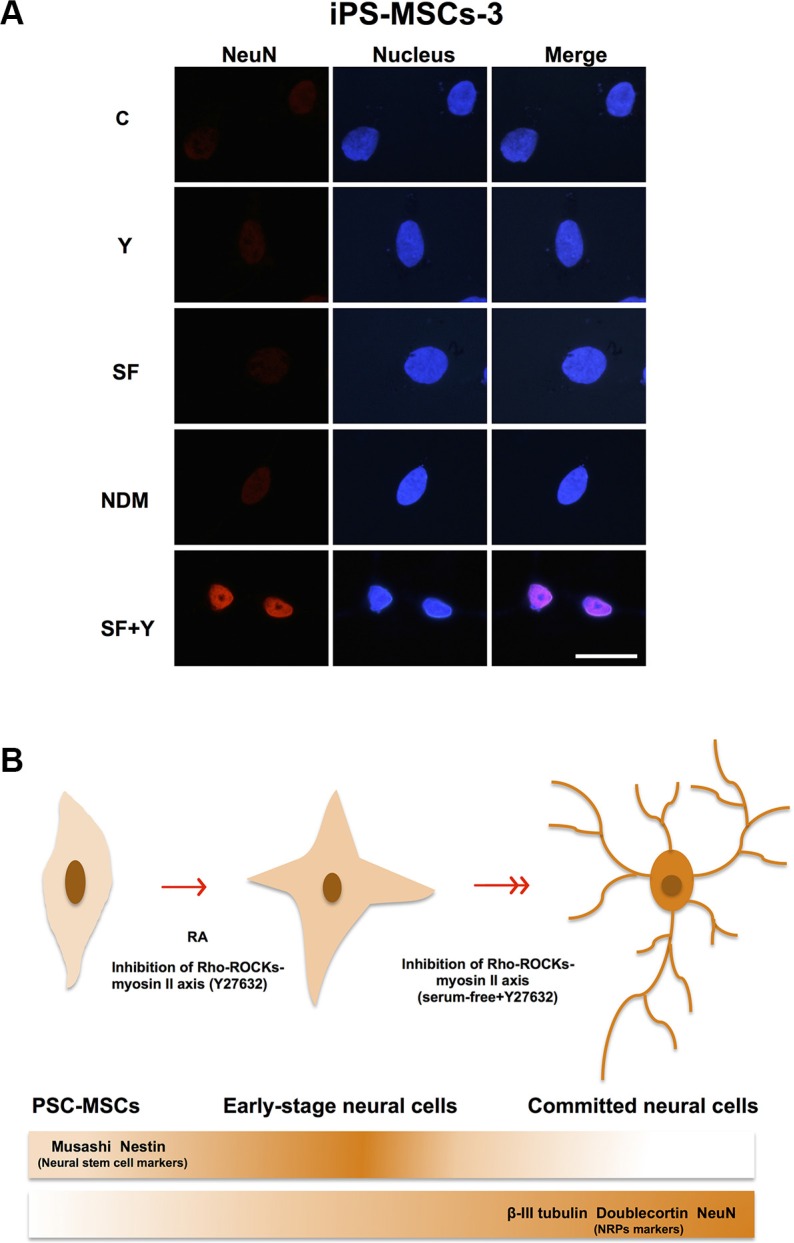
PSC-MSCs have differentiation capacity of neural lineage more than BM-MSCs (**A**) IF staining for protein expression of NeuN (red fluorescence) PSC-MSCs cultured in SF+Y. Nuclei were be detected by Hoechst 33342 (blue fluorescence). Scale bar: 20 μm. (**B**) PSC-MSCs express higher baseline levels of NSC genes *Nestin* and *Musashi* than BM-MSCs. Culturing of PSC-MSCs in NDM or with inhibition of ROCK-myosin II axis leads to neural lineage commitment. When ROCK inhibition of PSC-MSCs is performed under SF conditions, these progenitors can further differentiate into more committed neural cells.

NRPs are positive for expression of β-III-tubulin, Doublecortin and NeuN, but negative for NSC markers such as Nestin [26, 27]. β-III-tubulin, also known as Tuj-1, is a neuron-specific tubulin whose expression is increased during neuronal development [28]. Doublecortin is a microtubule-associated protein, which is required for neuronal migration into the cerebral cortex [29]. NeuN (Neuronal Nuclei), a neuron-specific nuclear protein, regulates RNA splicing and marks postmitotic neuronal cells [30]. Using IF staining, we found that of all the differentiation conditions, SF+Y resulted in the most apparent decreases in Nestin expression and concomitant increases in β-III-tubulin and Doublecortin expression (Figure 4A–4B). Interestingly, these changes in protein expression could only be seen in PSC-MSCs with committed neural cell morphology—cytoplasmic elongation, multiple dendritic process, and nuclear condensation—but not in PSC-MSCs that still retain undifferentiated, fibroblastic—typical MSC—cell morphology. Importantly, NeuN expression could also be detected in the nucleus of neural lineage-committed PSC-MSCs, which is strongly suggestive of further commitment past the NSC stage and along the neural lineage for these MSCs (Figure 5A). Taken together, our data indicate that PSC-MSCs could differentiate into more committed neural cells through modulation of the ROCK-myosin II axis in a SF environment (Figure 5B).

## DISCUSSION

Increasing evidence for MSC plasticity has brought hope for use of these versatile somatic stem cells for a multitude of clinical indications [6]. However, these rare progenitors are isolated from post-natal tissues, in which senescence rapidly sets in during *in vitro* expansion for therapeutic use [7, 31]. Human PSC-MSCs including ES-MSCs and iPS-MSCs, are capable of multilineage paraxial mesodermal differentiation as well as immunomodulation, similar to adult BM-MSCs [12, 32]. Additionally, PSC-MSCs are highly proliferative and renewable without the worry of teratoma formation inherent to its parental cell type [10, 11]. Our data demonstrates that PSC-MSCs have strong capacity to undergo neural differentiation, an extra-paraxial mesodermal lineage. Even in an undifferentiated state, PSC-MSCs expresses significantly higher levels of NSC markers such as Nestin and Musashi than adult BM-MSCs. Moreover, in complete medium—the standard medium for culturing all types of MSCs—inhibition of the ROCK-myosin II axis in PSC-MSCs increases expression of Nestin. When ROCK inhibition is applied under more standard neurogenic differentiation conditions (SF medium), PSC-MSCs further differentiate into a more committed neural phenotype, as demonstrated by reduction of nuclear area, nuclear NeuN protein expression, and expression of more committed NRP-proteins β-III-tubulin and Doublecortin. Our data therefore demonstrate that PSC-MSCs have the capacity to undergo significant neural lineage commitment. Ongoing studies are underway to ascertain the functionality of these PSC-MSC-differentiated neural-like cells in *in vivo* disease models for future clinical applications.

Cytoskeletal rearrangement has increasingly been shown to be a critical component during neural development. The proteins of RhoA family are known to be involved in neuronal cytoplasmic extension and growth of dendritic processes seen during the development of the central nervous system [16, 17, 33]. At the stage of neurite initiation, F-actin polymerization in the growth cone results in the appearance of actin-rich lamellipodium [34]. MAP2 and Doublecortin, which we found to be increased in PSC-MSCs after suppression of the ROCK-myosin II axis, are involved in mediating neuronal morphological changes via regulation of the cytoskeleton [35, 36]. Indeed, the severe neuronal phenotype found in Doublecortin-knockout mice is due to actin dysregulation [37]. The stage-specific importance of the cytoskeleton in neural development can be seen in our *in vitro* studies in which ROCK inhibition of PSC-MSCs in SF conditions—rather than with serum in complete medium—results acquisition of a more committed neural phenotype that is more profound than that induced with RA in SF medium. Our findings in this study are in line with other *in vitro* studies, in which inhibition of ROCK promotes neurite outgrowth in rat embryo hippocampal neurons and neuronal differentiation in mouse NSCs and ESCs [38, 39]. The importance of cell polarity during *in vitro* modeling of human PSC neural differentiation has also recently been shown in human iPSCs [40]. Moreover, recent reports also showed that ROCK inhibition could accelerate the process of direct reprogramming of fibroblasts into neurons [41]. These studies and ours further confirm the important role of the RhoA family and cytoskeletal rearrangement in MSC neural commitment, and demonstrate the utility of *in vitro* recapitulation of *in vivo* information.

While iPSCs and direct reprograming of fibroblasts can generate NRPs or other neural cells, these two cellular sources are not without its problems. For iPSCs, unless complete differentiation of all cells is achieved, teratoma formation is always a concern; the use of PSC-MSCs abrogates this possibility. On the other hand, direct reprograming of fibroblasts to neural cells is highly inefficient as not to be clinically relevant currently [42]. In comparison to other adult/post-natal source MSCs such as BM-MSCs, PSC-MSCs possess a stronger capacity for neural differentiation; we speculate that this may be due to the early developmental stage of these MSCs being derived directly from PSCs, since it has been demonstrated that developmentally early stage MSCs isolated from fetal tissue can have broader differentiation capacity than adult BM-MSCs [43]. One possible mechanism accounting for these functional differences may be the higher expression levels of neural developmentally relevant genes such as Musashi in PSC-MSCs compared to BM-MSCs (Figure 1C). During neural development, Musashi, a translational regulator, represses *m-Numb* mRNA translation and activate Notch signaling [44]. This results in the maintenance of self-renewal and differentiation potential in NSCs [45]. Further studies would be necessary to confirm whether these early neural developmental specifiers are more active in PSC-MSCs compared to adult/post-natal MSCs.

## MATERIALS AND METHODS

### Cell culture

PSC-MSCs were derived and characterized as previously reported [10]. ES-MSCs were derived from HSF-6 (University of California, San Francisco) and H1 (Wisconsin Alumni Research Foundation) [46, 47]. iPS-MSCs were derived from iPS which were generated by two methods: (1) Sendai Virus induction (Sendai Reprogramming Kit; Invitrogen-Thermo Fisher Scientific, MA, USA) with all four Yamanaka factors into human peripheral blood mononuclear cells as per manufacturer's and published protocols [48] for iPS-MSC clones 1 and 2; and (2) lentiviral induction with Oct-4 and Sox-2 into human umbilical vein endothelial cells (Bioresource Collection and Research Center, Hsinchu, Taiwan) as previously reported [49] for iPS-MSC clone 3. BM-MSCs were obtained commercially (Promocell, Heidelberg, Germany) and cultured as previously described [4]. All MSCs were cultured with complete medium consisting of DMEM-low glucose supplemented with 1% penicillin/streptomycin, 1% L-glutamine (all from Gibco-Thermo Fisher Scientific), and 10% fetal bovine serum (FBS, selected lots from Hyclone-Thermo Fisher Scientific) and expanded as previously described [10].

### Differentiation studies

PSC-MSCs and BM-MSCs were characterized for trilineage paraxial mesodermal differentiation capacity as previously described [10, 50]. For neurogenic differentiation, cells were cultured on six-well plates (5000 cells/cm^2^) and treated with 0.5 μM retinoic acid (RA; Sigma-Aldrich, St. Louis, MO, USA) for 48 hours in serum-free medium [51]. The ROCK inhibitor Y-27632 and blebbistatin (10 μM, both from Sigma-Aldrich) was added to either complete medium or serum-free medium for 48 hours as indicated [15].

### Immunofluorescent (IF) staining

IF staining was performed as previously reported [15]. Briefly, cells were fixed with 4% paraformaldehyde and permeabilized with 0.1% Triton-X 100 (Sigma-Aldrich) for 20 minutes. Cells were then blocked with 5% BSA then incubated with primary antibodies against Nestin (1:100; mouse monoclonal, Millipore-Merck, Darmstadt, Germany), MAP2 (1:100; rabbit polyclonal, Millipore-Merck), β-III-tubulin (1:100; rabbit polyclonal, Abcam, Cambridge, MA, USA), Doublecortin (1:100; rabbit polyclonal, Abcam), or NeuN (1:100; mouse monoclonal, Millipore-Merck) at room temperature for two hours. Cells were washed with phosphate-buffered saline (PBS) and incubated with FITC-conjugated or PE-conjugated secondary antibodies (1:200; Santa Cruz Biotechnology, Santa Cruz, CA, USA) at room temperature for 2 hours. Nuclei were stained with Hoechst 33342 (Sigma-Aldrich). F-actin was detected by rhodamine-conjugated phalloidin (1:200; Invitrogen-Thermo Fisher Scientific). A fluorescence microscope was used for visualization (Olympus, Tokyo, Japan).

### Real-time quantitative polymerase chain reaction (real-time PCR)

Real-time PCR was performed as previously reported [15]. Total RNAs were extracted with TRIzol Reagent (Invitrogen-Thermo Fisher Scientific) and cDNA was synthesized from RNA using RevertTra Ace cDNA Synthesis Kit (Toyobo, Osaka, Japan). Real-time PCR was performed by using Fast SYBR^®^ Green Master Mix qPCR Kit on the ABI 7500 Real-Time PCR System (both from Applied Biosystems-Thermo Fisher Scientific). Relative gene expression levels were analyzed as indicated by the manufacturer. Primers used are shown in Table 1.

**Table 1:** Primer list

Gene		Primer 5′-3′
Nestin	F	AACAGCGACGGAGGTCTCTA
	R	TTCTCTTGTCCCGCAGACTT
MAP2	F	CCAGGTGGCGGACGTGTGAA
	R	GCCACGCTGGATCTGCCTGG
Musashi-1	F	GTCTCGAGTCATGCCCTACG
	R	GCAGTGAGAGGAATGGCTGT
GAPDH	F	GTGGACCTGACCTGCCGTCT
	R	GGAGGAGTGGGTGTCGCTGT

F, forward primer; R, reverse primer.

### Statistics

All data were expressed as mean ± SEM. Statistical significance between two samples was analyzed by the Student's *t* test. ANOVA was used to analyze the significance between multiple samples. A value of *p* < 0.05 was defined as statistical significance.

## CONCLUSIONS

PSC-MSCs show potent capacity to differentiate into a committed neural phenotype with high expression of NSC genes at baseline. Cytoskeleton rearrangement through inhibition of the ROCK-myosin II axis in a neural differentiating environment can strongly induce PSC-MSCs to acquire a committed neural cell phenotype, with reduction of nuclear size and upregulation of late neural lineage-specific markers including β-III-tubulin, Doublecortin, and nuclear NeuN. Our data gives support to the use of PSC-MSCs as a possible candidate progenitor cell in therapeutic application towards neuro-degenerative and related diseases, as well as highlight the importance of the cytoskeleton in neural differentiation.
